# Whole-Body Cryostimulation in Complex Regional Pain Syndrome: A Case Study

**DOI:** 10.3390/jcm15062142

**Published:** 2026-03-11

**Authors:** Paolo Piterà, Alberto Camedda, Elisa Prina, Eleonora Franzini Tibaldeo, Gabriele Baccalaro, Paolo Capodaglio

**Affiliations:** 1 Department of Neurosciences “Rita Levi Montalcini”, University of Turin, 10126 Turin, Italy; e.prina@auxologico.it; 2 Laboratory of Clinical Neurobiology, IRCCS Istituto Auxologico Italiano, San Giuseppe Hospital, 28824 Verbania, Italy; 3 Division of Neurology and Neurorehabilitation, IRCCS Istituto Auxologico Italiano, 28824 Verbania, Italy; albertocame95@gmail.com; 4 Department of Surgical Sciences, Physical and Rehabilitation Medicine, University of Torino, 10121 Torino, Italy; e.tibaldeo@auxologico.it; 5 Research Laboratory in Biomechanics, Rehabilitation and Ergonomics, IRCCS Istituto Auxologico Italiano, San Giuseppe Hospital, Piancavallo, 28824 Verbania, Italy; g.baccalaro@auxologico.it (G.B.); p.capodaglio@auxologico.it (P.C.); 6 Department of Biomedical, Surgical and Dental Sciences, University of Milan, 20122 Milan, Italy

**Keywords:** Complex Regional Pain Syndrome (CRPS), Whole-Body Cryostimulation (WBC), chronic pain, non-pharmacological intervention, rehabilitation, pain management

## Abstract

**Background/Objectives:** Complex Regional Pain Syndrome (CRPS) is a debilitating pain condition with complex pathophysiology and limited treatment efficacy. Whole-body cryostimulation (WBC) has shown promising results in other chronic pain syndromes, but no studies to date have examined its use in CRPS. To evaluate the safety, feasibility, and potential benefits of WBC in a female patient with CRPS of the ankle. **Methods**: A 65-year-old female outpatient with type I CRPS at the right ankle underwent 15 WBC sessions (3 min at −110 °C) over two weeks, without any concurrent pharmacological or rehabilitative interventions. Assessments at baseline and post-intervention included standardized measures of pain (VAS, SF-MPQ), disability (PDI), catastrophizing (PCS), mobility (TUG, Chair Stand Test), strength and ROM (goniometry, MRC), psychosocial status (SF-36, WHO-5, PSQI, BDI, STAI), and MRI of the right knee and ankle. **Results**: Post-treatment, the patient showed substantial improvements in pain (VAS −66.7%, SF-MPQ −51.7%), function (TUG −31.8%), muscle strength, psychological well-being, and quality of life. MRI and edema measurements indicated stabilization or regression of inflammatory features. No adverse effects were reported. **Conclusions**: This case suggests that WBC may represent a safe, well-tolerated, non-pharmacological intervention for CRPS, with potential to improve pain, function, and well-being.

## 1. Introduction

Complex regional pain syndrome (CRPS) is a chronic pain disorder that typically affects one or more extremities, characterized by severe, disproportionate pain accompanied by a combination of sensory, autonomic, motor, and trophic disturbances beyond what the initial trauma would explain [1,2]. CRPS has been subdivided into type I, reflex sympathetic dystrophy, which occurs after an illness or injury that did not directly damage the nerves, and type II, causalgia, which occurs after a distinct nerve injury [3]. Diagnostic criteria (such as the Budapest criteria) require the presence of symptoms and signs in categories including sensory (allodynia or hyperalgesia), vasomotor (skin color or temperature changes), sudomotor/edema (swelling or sweating changes), and motor/trophic (weakness, tremor, hair/nail changes), with no alternate diagnosis better accounting for them [1,4,5]. CRPS is relatively uncommon, with incidence estimates ranging from about 5.5 to 26 per 100,000 person-years [6] (higher in middle-aged women and following fractures). Nonetheless, its impact is profound: CRPS pain is ranked among the most intense of any medical condition [7], and patients suffer substantial functional impairment and disability [8]. The prolonged pain and physical limitations frequently give rise to psychological distress, as depression, anxiety, underscoring the high burden of the disease and the urgent need for effective interventions [1].

The multifactorial nature of CRPS, involving nociceptive, inflammatory, autonomic, and psychological dimensions, poses a major challenge for management and often necessitates a multidisciplinary treatment approach [9,10]. Conventional therapy for CRPS is primarily supportive and symptomatic, with important limitations in efficacy. Standard care typically combines rehabilitative interventions and pharmacotherapy aimed at reducing pain and inflammation [11]. Parenteral bisphosphonates and short-course corticosteroids represent the most evidence-based pharmacological options for early-stage CRPS-I [12]. In contrast, agents such as gabapentinoids or SNRIs are occasionally used in chronic cases, although their efficacy remains modest and evidence limited [2,13]. For refractory presentations, interventions like sympathetic nerve blocks or intravenous ketamine infusions have also been trialed, but their application is often constrained by invasiveness and potential adverse effects [14].

Rehabilitation is a cornerstone of CRPS treatment: sustained physical and occupational therapy, often combined with techniques like mirror therapy or graded motor imagery, is employed to restore limb function and prevent disuse [5,15]. Psychological support (for instance, cognitive–behavioral therapy addressing pain-related fear and coping) is also recommended, given the influence of psychosocial factors on CRPS outcomes [16].

Despite these varied treatments, outcomes are often suboptimal with many patients experiencing only partial pain relief and remaining functionally limited [17]. Conventional medications may provide insufficient benefit or induce significant adverse effects, and the syndrome can become chronic [1].

This therapeutic difficulty has prompted interest in novel adjunctive therapies that can more effectively target pain, inflammation, and central sensitization underlying CRPS [18].

Whole-body cryostimulation (WBC), the therapeutic exposure of the entire body to extremely cold air (typically −110 °C to −140 °C for 2–3 min), is emerging as a promising non-pharmacological treatment for various chronic pain and inflammatory conditions [19]. WBC exerts analgesic and anti-inflammatory effects through peripheral vasoconstriction, neuroendocrine activation and modulation of immune and pain pathways [20,21,22,23]. In addition to its direct physical effects, WBC may confer psychological benefits: patients often report reduced fatigue, elevated mood, and better sleep after a course of cryotherapy, with some studies reporting improvements in depressive symptoms and overall quality of life [19,20,24].

In CRPS, pain chronicity is primarily driven by central sensitization, neurogenic inflammation, and altered pain modulation pathways, as highlighted by recent meta-analyses of quantitative sensory testing that reveal generalized hyperalgesia and reduced inhibitory control [25]. Within this framework, WBC may represent a promising therapeutic adjunct, as its repeated application has been shown to reduce pain severity [26,27], lower pro-inflammatory cytokine levels [28] and improve functional capacity in other chronic pain conditions [29]. By potentially mitigating peripheral and central sensitization and reducing inflammatory mediators [24,30], WBC could contribute to pain relief and functional restoration in CRPS, offering a non-pharmacological option aligned with the complex pathophysiology of the syndrome.

In conditions characterized by central sensitization and chronic inflammation such as fibromyalgia and rheumatoid arthritis, repeated WBC sessions led to reductions in pain perception and disease activity along with enhancements in physical performance and mood [19,23,31,32]. These findings suggest that WBC can favorably influence pain, inflammation, and even psychological well-being in a wide range of chronic clinical conditions, with minimal risk.

Given this background, WBC presents a compelling theoretical approach to address the multifaceted pathology of CRPS. The global anti-inflammatory and neuromodulatory effects of extreme cold exposure might counteract the persistent inflammation and sensitization seen in CRPS, while the associated improvements in pain, mobility, and mood could synergistically enhance patient function and quality of life. However, despite its successful application in other chronic pain syndromes, WBC has not yet been studied in patients with CRPS.

**Figure 1:**
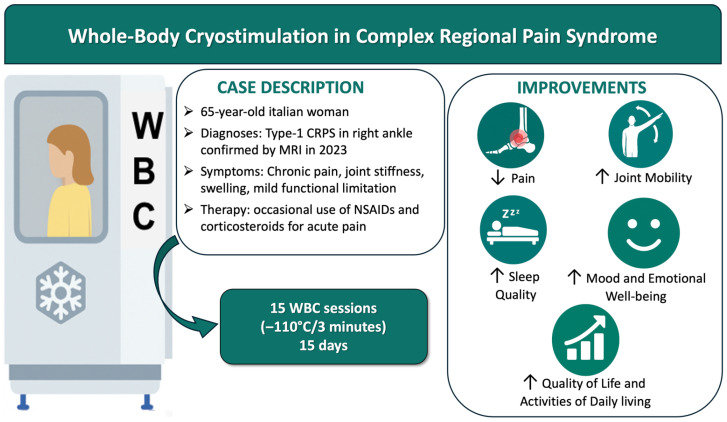
Overview of the study design and main outcomes.

Here, we present what is (to the best of our knowledge) the first documented case of a CRPS patient treated with WBC as an adjunct to conventional rehabilitation. The patient, suffering from longstanding type 1 CRPS, underwent a protocol of fifteen WBC sessions (3 min at −110 °C per session) without any other concurrent rehabilitative intervention. In this report, we describe the patient’s outcomes in terms of chronic pain severity, functional improvement, quality of life, and psychological status following the inclusion of WBC. A schematic overview of the case, the WBC intervention protocol, and the main clinical outcomes is presented in Figure 1.

## 2. Materials and Methods

### 2.1. Case Description and Clinical History

A 65-year-old female Caucasian outpatient was recruited at the San Giuseppe Hospital (IRCCS Istituto Auxologico Italiano, Piancavallo, Italy) to participate in an experimental research protocol investigating the potential effects of WBC on CRPS.

As this case report was designed to explore the effects WBC on an individual with CRPS, no additional inclusion criteria were applied beyond the diagnosis of CRSP, reflecting the exploratory nature of the study. The exclusion criteria were limited to the contraindications specific to WBC [33], which includes severe cardiovascular and respiratory conditions, peripheral vascular disease, acute infections, pregnancy, and significant psychiatric or cognitive disorders. Each criterion was thoroughly assessed to ensure the patient’s eligibility for the intervention.

The patient reported the onset of pain in the right ankle in August 2023, initially accompanied by localized swelling on the lateral side. She could not recall any specific acute triggering event. She has been and currently still is employed in a cleaning company with frequent manual handling tasks in a hospital setting and carrying loads along long hospital corridors. Over the following months, both the intensity of pain and the degree of swelling progressively increased, ultimately affecting her ability to perform daily activities. Despite this, she continued her occupational tasks, although at a slower pace and with more frequent pauses.

The diagnosis of Complex Regional Pain Syndrome (CRPS) was confirmed in 2024 according to the Budapest diagnostic criteria. The patient was not prescribed any physical therapy, consistent medications or infiltrative treatments. Pain management with non-steroidal anti-inflammatory drugs (NSAIDs) or corticosteroids (Deltacortene) was limited to the episodes of exacerbation. 

This single-patient case report explores the potential effects of WBC as an adjunctive treatment in a female patient diagnosed with Complex Regional Pain Syndrome (CRPS) type I of the right ankle. The intervention consisted of 15 WBC sessions administered over two weeks, each involving a 3 min exposure at −110 °C in a certified cryochamber under medical supervision. Sessions were performed once daily, with additional sessions on selected days during the second week, resulting in a total of 15 exposures. The patient was referred to the cryotherapy laboratory specifically to undergo a cycle of WBC and did not receive any concurrent pharmacological treatment, physiotherapy, or other rehabilitative interventions during the observational period.

This case report was conducted in accordance with the ethical principles of the Declaration of Helsinki and was approved by the Ethics Committee of Istituto Auxologico Italiano (protocol code: 2021_05_18_14). The patient received full information about the purpose and procedures of the study and provided written informed consent for participation and for the publication of anonymized clinical data.

### 2.2. Clinical Examination

On clinical examination, the patient presented with persistent pain primarily localized in the right ankle and right knee. The pain was described as pounding, burning, and cramping, often perceived as piercing and squeezing, accompanied by tingling sensations and a subjective perception of coldness or deep aching. These descriptors were reflected in high scores across multiple categories of the McGill Pain Questionnaire (PRI total: 60/78). The patient reported worsening of symptoms in response to mechanical load, weather or temperature changes, physical exertion, and emotional stressors, especially those linked to returning to work or activity in cold environments.

During flare-ups, the pain was described as unbearable, significantly interfering with daily functioning, emotional regulation, and sleep quality. Nocturnal pain was particularly debilitating, often associated with distress. Despite a long clinical history and multiple therapeutic attempts, including medications and various physical therapies, the patient always reported minimal benefit, contributing to a persistent sense of helplessness, frustration, and catastrophic thinking. She emphasized that the pain extended beyond the physical domain, deeply affecting her mental and emotional well-being.

### 2.3. Symptoms Assessment

Clinical, functional, and psychological evaluations were performed at baseline (T0), just before the beginning of the WBC cycle, and just after the completion of the WBC treatment cycle (T1) allowing the calculation of percentage changes in performance.

All assessments were conducted under standardized conditions by the same clinical and research team to minimize variability. Specifically, all functional and clinical evaluations were performed by a licensed physiotherapist (A.C.) with expertise in chronic pain rehabilitation, ensuring consistency and reliability in measurement procedures.

#### 2.3.1. Pain and Pain-Related Conditions

Pain assessment was performed using multiple complementary tools. Pain-related outcomes were assessed using a standardized battery of validated questionnaires. Pain intensity and qualitative pain characteristics were evaluated using the Short-Form McGill Pain Questionnaire (SF-MPQ) [34,35], a multidimensional instrument that evaluates both the sensory and affective qualities of pain through a list of descriptors, and with the Visual Analog Scale (VAS) [36,37,38]. In addition, the Pain Numeric Rating Scale (PNRS) [38] was used as a rapid and user-friendly tool for quantifying perceived pain on a discrete scale from 0 (“no pain”) to 10 (“worst imaginable pain”). While both VAS and PNRS assess pain intensity, they differ in format and sensitivity: VAS captures more subtle changes on a continuous scale, whereas PNRS is easier to administer and interpret in clinical settings. The inclusion of both tools allowed for a more robust assessment of pain perception, combining sensitivity and clinical practicality. Pain-related functional interference was assessed with the Pain Disability Index (PDI) [39], which evaluates the extent to which pain interferes with daily activities across multiple life domains, while maladaptive cognitive and emotional responses to pain were evaluated using the Pain Catastrophizing Scale (PCS) [40], which measures catastrophic thinking related to pain, including rumination, magnification, and helplessness.

#### 2.3.2. Functional Mobility and Performance

Functional mobility and motor performance were assessed through a standardized battery of clinical tests, conducted under controlled conditions by an experienced physiotherapist specialized in chronic pain rehabilitation. The Timed Up and Go (TUG) [41] test was used to evaluate dynamic mobility, balance, and fall risk: the patient was instructed to rise from a standard chair, walk three meters, turn, return, and sit down, while the total time was recorded in seconds. Lower-limb strength and functional capacity were measured using the 30-Second Chair Stand Test (30SCT) [42], in which the patient performed the maximum possible number of sit-to-stand repetitions within thirty seconds.

#### 2.3.3. Range of Motion (ROM) and Strength

Joint mobility and muscle strength were assessed using standardized clinical methods [43]: Passive Range of Motion (PROM) and Active Range of Motion (AROM) were evaluated using a standard manual goniometer (in degrees) at the knee (flexion, extension), and ankle (dorsiflexion, plantarflexion). PROM was defined as the maximal joint excursion achieved by the examiner without patient muscle activation [43], while AROM referred to the maximal range achieved through voluntary muscle contraction [43]. Muscle strength was assessed using the Oxford Scale (also referred to as MRC score) [44]. Strength testing focused on the knee (flexion, extension) and ankle (dorsiflexion, plantarflexion).

#### 2.3.4. Skin Temperature Assessment

Skin temperature was measured bilaterally at the right and left ankle using a high-precision infrared thermometer (Fluke 62 Max+, Fluke Corporation, Everett, WA, USA), featuring an accuracy of ±1 °C or ±1% of the reading and a distance-to-spot ratio of 12:1, with a measurement range from −30 °C to +650 °C. Measurements were collected at a standardized distance and perpendicular to the skin surface. Skin temperature was recorded within 1 min immediately before and after each WBC session, allowing the assessment of the acute thermal response to cryostimulation throughout the intervention period.

#### 2.3.5. Edema Assessment

Ankle circumference was measured using a non-elastic tape measure on both limbs to assess peripheral swelling, applying the standardized figure-of-eight method described for ankle girth assessment [45]. Measurements were performed in standardized conditions immediately before the first WBC session (T1) and after the final session (T15), to capture cumulative volumetric changes. Although this method does not differentiate between edema subtypes, the figure-of-eight technique is widely accepted as a reliable and reproducible procedure for monitoring ankle soft-tissue volume in clinical settings.

#### 2.3.6. Psychosocial Assessment

We assessed health-related quality of life using the Short Form Health Survey 36 (SF-36), which evaluates multiple domains of physical and mental health [46]. Sleep quality was measured with the Pittsburgh Sleep Quality Index (PSQI) [47], a questionnaire assessing sleep quality and disturbances. Fatigue was evaluated with Fatigue Severity Scale (FSS) [48], a 9-item scale measuring the impact of fatigue on daily functioning. To assess overall subjective well-being, we utilized the five-item World Health Organization Well-Being Index (WHO-5) [49]. The severity of depression was assessed with the Beck Depression Inventory (BDI) [50], a 21-item self-report questionnaire, while anxiety was assessed using the State–Trait Anxiety Inventory (STAI), which measures both state (STAI-Y1) and trait (STAI-Y2) anxiety through two 20-item subscales [51]. Psychosocial measures, including the SF-36, WHO-5, PSQI, BDI, and STAI, were selected for their validated reliability and relevance in evaluating the intervention’s impact on multidimensional quality of life, subjective well-being, and sleep quality.

#### 2.3.7. Magnetic Resonance Imaging

To evaluate the structural joint condition before and after the WBC intervention, the patient underwent three separate MRI assessments focused on the right knee and right ankle. The first MRI was performed in August 2024, approximately one year before the start of the intervention, providing a baseline for chronic degenerative joint status. A second MRI was acquired on 7 July 2025, immediately prior to the 15-session WBC protocol, and the final imaging was completed on 19 August 2025, within one week after completing the intervention.

All MRIs were performed using high-field (1.5 T) whole-body scanners (Ingenia, Philips Healthcare, Amsterdam, The Netherlands), employing multiplanar and multiparametric sequences to examine bone, cartilage, synovial, ligamentous, and soft tissue structures in detail. The imaging protocols were consistent across timepoints to ensure comparability. All MRI examinations were performed in an external radiology center independent from the study site. Image interpretation and reports were produced by certified radiologists as part of routine clinical practice. The radiologists were not involved in the study and were unaware of the research objectives and of the WBC intervention, thus ensuring that image interpretation was performed independently of the study hypothesis.

### 2.4. Intervention

#### Whole-Body Cryostimulation (WBC)

The WBC protocol consisted of 15 daily sessions administered at 12:00 p.m. from 28 July to 14 August 2025. Prior to treatment, the patient was screened for contraindications according to the 2025 position paper on contraindications to WBC issued by the WBC Working Group of the International Institute of Refrigeration [33]. A preliminary 1 min familiarization exposure at −110 °C was conducted in a nitrogen-cooled cryochamber (Arctic model, CryoScience, Rome, Italy), with the patient wearing minimal clothing and protections for the extremities, in accordance with WBC safety guidelines. She then underwent a cycle of 15 WBC sessions at −110 °C under the supervision of specifically trained operator in WBC procedures (P.P.). The initial plan consisted of one daily WBC session lasting 3 min (10-session WBC cycle), to be completed over two weeks. However, due to the patient’s tolerability and favourable clinical response, the protocol was adjusted during the second week to include double sessions on selected days, allowing the completion of 15 sessions within the same timeframe.

### 2.5. Statistical Analysis

Given the single-case design of this report, no inferential statistical analyses were performed. Clinical, functional, and psychosocial outcomes were summarized descriptively using absolute values and percentage changes between baseline (T0) and post-intervention (T1). Percentage changes (Δ%) were calculated to provide a simple quantitative representation of the magnitude of change across outcome measures. All data are presented descriptively to illustrate potential trends associated with the WBC intervention.

## 3. Results

### 3.1. Psychosocial Outcomes

Following the WBC cycle, the patient showed marked improvements across multiple psychosocial, functional, and pain-related domains. Quality of life, as assessed by the SF-36, improved in almost all subscales, with the most substantial changes observed in role limitations due to emotional problems (+33%), general health (+30%), and emotional well-being (+28%). WHO-5 scores nearly doubled (+88.9%), reflecting enhanced psychological well-being, while fatigue (FSS: −46%) and sleep quality (PSQI: −44.4%) also improved significantly. Depressive (BDI: −16.7%) and anxiety symptoms (STAI-Y1: −27.7%, STAI-Y2: −15.7%) decreased. In terms of pain and disability, the patient reported substantial reductions across all domains: PRI SF-MPQ (−51.7%), VAS (−66.7%), PNRS (−70%), and catastrophizing (PCS: −73.5%), with a milder reduction in perceived disability (PDI: −15.6%). Detailed outcomes are presented in Table 1.

Significant reductions in pain intensity, pain-related disability, and maladaptive pain cognitions were observed following the WBC sessions. These improvements, reflected by decreases in PRI SF-MPQ (−51.7%), VAS (−66.7%), PNRS (−70%), PDI (−15.6%), and PCS (−73.5%), indicate a substantial attenuation of both the sensory and affective dimensions of pain. These changes, evaluated through targeted clinical and functional assessments, are summarized in Table 2.

### 3.2. Functional Mobility and Motor Performance

Following the WBC intervention, the patient showed significant improvements in functional mobility (depicted in Table 3). The Timed Up and Go (TUG) test time decreased by 31.8%, indicating enhanced dynamic mobility and gait efficiency. Similarly, lower-limb strength and endurance improved, as shown by a 25% increase in the 30-Second Chair Stand Test (30SCT) repetitions.

### 3.3. Range of Motion (ROM) and Strength

Following the 15-session WBC protocol, the patient demonstrated clinically meaningful improvements in both range of motion (ROM) and muscle strength, particularly in the affected lower limb.

In the lower limb, ankle dorsiflexion AROM showed a marked bilateral gain (+66.7%), while ankle plantarflexion AROM increased by +60% on the right and +14.3% on the left.

Strength assessments reflected similar positive trends: knee flexion MRC increased from 3 to 4 on both sides (+33.3%).

Overall, these findings suggest that WBC may have contributed to both mobility restoration and muscle strength recovery, potentially enhancing functional performance. The results are depicted in Table 4.

### 3.4. Temperature and Edema

Ankle edema measurements showed a slight reduction following the WBC cycle. At baseline (T0), the right ankle circumference measured 53 cm, which decreased to 51 cm at T1 (−2 cm), while the left ankle remained stable at 51 cm. This localized reduction in swelling, particularly on the right side, may reflect a mild anti-edematous effect of cryostimulation in chronic musculoskeletal conditions.

Regarding temperatures, baseline (session 1), skin temperature was higher in the right ankle (35.2 °C) compared to the left ankle (32.0 °C), suggesting potential local differences in vascular tone or inflammatory activity. Throughout the 15-session WBC protocol, both ankles exhibited consistent and substantial reductions in skin temperature after each exposure, with an average decrease of approximately 9.2 °C. This pattern was maintained across all sessions without significant fluctuations, indicating a stable thermoregulatory response to cryostimulation. Temperature results are reported in Table 5.

Interestingly, during the initial WBC sessions, the affected ankle (right side) exhibited a markedly greater pre–post cooling delta compared to the contralateral side, likely due to its elevated baseline temperature and increased local inflammation. However, as the 15-session protocol progressed, the right ankle’s baseline temperature gradually decreased and aligned closely with that of the left ankle. This convergence in both pre-cooling values and thermal response deltas suggests a progressive attenuation of local inflammatory processes and a restoration of peripheral thermal homeostasis, supporting the anti-inflammatory effect of WBC in the affected region.

### 3.5. Magnetic Resonance Imaging

The comparative analysis of magnetic resonance imaging (MRI) findings conducted across three timepoints—August 2024, July 2025 (pre-WBC), and August 2025 (post-WBC)—provides a valuable perspective on the structural evolution of the patient’s musculoskeletal condition over time and in response to the WBC protocol. The earliest MRI scan, performed in 2024, documented a constellation of chronic degenerative alterations, including medial meniscal extrusion and thinning, early chondral wear at knee level and subchondral bone signal changes, soft-tissue edema in the right ankle, particularly in the dorsal region. These findings were indicative of an ongoing degenerative and inflammatory process, likely contributing to the patient’s pain, functional limitation, and localized swelling.

In July 2025, before the initiation of the WBC intervention, imaging revealed a modest progression of the degenerative picture, with persistent alterations in the knee and ankle joints. In the knee, a radial tear of the medial meniscus was identified alongside grade II tibiofemoral chondropathy, mild synovitis, and early cartilaginous thinning, yet without joint effusion or significant osteophyte formation. In the ankle, the presence of periastragalic soft tissue edema, edema of the sinus tarsi, and narrowing of the joint space suggested a chronic inflammatory involvement of the tibiotarsal and midfoot joints, with early signs of arthrosis and persistent subchondral bone alterations.

Following the 15-session WBC protocol, the final MRI scan conducted in August 2025 revealed a qualitative reduction in inflammatory and edematous signs. In the knee, the synovial reaction appeared diminished, and there was no evidence of joint effusion or progressive cartilage degeneration. The pre-existing meniscal and ligamentous conditions remained unchanged. In the ankle, there was a noticeable reduction in soft tissue edema, with improved subchondral bone appearance and no worsening of the pseudo-cystic areas or joint space narrowing. The retrotibial and peroneo-astragalic regions showed decreased signal intensity in the sequences previously associated with inflammatory processes, suggesting a possible reduction in local inflammatory activity.

MRI findings, reported in Table 6, are summarized from the original radiological reports and are presented descriptively to allow a qualitative comparison across timepoints.

## 4. Discussion

To the best of our knowledge, this case study represents the first documented application of WBC in a patient with CRPS type I. The intervention was associated with clinically relevant improvements in multiple domains, including pain intensity and affective response, functional mobility, psychological well-being, and health-related quality of life. These improvements were observed in the absence of any concomitant pharmacological or physiotherapeutic treatment, reinforcing the hypothesis that WBC itself may exert meaningful therapeutic effects in CRPS. Pain reduction was one of the most striking outcomes. Following the 15-session WBC protocol, the patient reported substantial improvements across all pain-related assessments. These findings are in line with previous reports of WBC efficacy in other chronic pain syndromes such as fibromyalgia [52], osteoarthritis, and rheumatoid arthritis, where WBC was shown to decrease central sensitization, modulate nociceptive pathways, and reduce systemic inflammation [29,53]. The consistency of these results across both sensory and cognitive–affective pain domains underscores WBC’s potential role in neuromodulation and anti-inflammation in complex chronic pain conditions. The patient demonstrated functional improvements in mobility and strength. The decrease in TUG time and the increase in chair stand repetitions reflect gains in dynamic mobility, postural control, and lower limb endurance, suggesting that WBC may help restore motor performance and reduce disuse-related decline in CRPS. The improvements observed in functional mobility and performance may also be partially explained by the marked reduction in pain intensity following the intervention. Pain reduction likely facilitated greater use of the affected limb during daily activities throughout the treatment period, thereby promoting increased mobility and functional engagement. This mechanism is consistent with the well-established relationship between pain relief, enhanced movement, and subsequent improvements in physical performance in chronic pain conditions [54,55].

Furthermore, increases in active range of motion (AROM) and manual muscle testing scores (Oxford dynamometry, provide preliminary support for a broader restorative effect of WBC on musculoskeletal function. These motor outcomes may be partially attributable to pain relief, but also suggest a potential influence on muscle tone regulation, proprioceptive integration, and inflammation-induced joint stiffness, as previously hypothesized in the cryotherapy literature [56,57,58]. Psychological and psychosocial domains also showed notable improvement. WHO-5 scores nearly doubled, depressive and anxiety symptoms decreased, and SF-36 subscales related to general health, emotional well-being, and role functioning improved significantly. These changes are consistent with prior evidence indicating that WBC may exert positive effects on mood, fatigue, and sleep quality, possibly through mechanisms involving catecholamine release [59] improved autonomic regulation [60,61], and endorphin-mediated enhancement of well-being [31]. While these psychophysiological effects remain speculative in CRPS, they warrant further investigation given the central role of psychological distress in chronic pain persistence and disability. While we acknowledge that causality cannot be firmly established in a single-case design, the improvements observed in pain, function, and psychological well-being can be reasonably attributed to the WBC intervention, as no concurrent pharmacological or rehabilitative treatments were administered during the study period. However, the patient’s positive emotional response to the intervention may have contributed to the observed outcomes by enhancing expectancy-related mechanisms and activating placebo-responsive pathways. The novelty of the treatment, the perceived technological appeal of WBC, and the supportive therapeutic context in which the sessions were delivered may have further reinforced the patient’s belief in the efficacy of WBC, thereby amplifying the placebo response. While this does not diminish the clinical relevance of the improvements observed, it underscores the importance of cautious interpretation and the need for controlled studies to clarify the specific physiological effects of WBC from non-specific contextual or psychological influences. Potential confounders in this study include the natural variability of CRPS symptomatology over time, spontaneous fluctuations in pain and function, the lack of blinding or control condition, and the patient’s positive emotional expectations toward the intervention, which may have amplified placebo-related responses. To address these confounding factors, the study outcomes were measured immediately before and after the WBC cycle. Additionally, the consistency of the positive findings across multiple outcome measures, including subjective reports, clinical scales, and objective instrumental assessments, strengthens the validity of the observed improvements. Another key finding from this report is the observed stability or qualitative improvement in MRI-detected joint pathology. Although structural changes in such a short time frame should be interpreted cautiously, these imaging results support the hypothesis of a local anti-inflammatory effect of WBC, possibly mediated by reflex vasoconstriction-vasodilation cycles and systemic immune modulation. This aligns with studies reporting decreased levels of pro-inflammatory cytokines and oxidative stress markers after repeated WBC exposures in inflammatory conditions [62]. This case study has several limitations. The single-subject design and the brief duration of the intervention restrict the generalizability of the findings and preclude long-term outcome assessment. It is also important to note that the assessment of muscular strength presents intrinsic limitations in terms of accuracy and objectivity. Specifically, it was not possible to employ isokinetic dynamometry, which would have provided more sensitive and quantitative measurements of joint strength. Instead, the evaluation relied on the Medical Research Council (MRC) scale, a low-sensitivity tool that is dependent on the examiner’s clinical experience and may be less reliable in detecting subtle changes [63]. Despite these limitations, this study underscores the innovative potential of WBC as a restorative, non-pharmacologic, and non-invasive intervention for the management of CRPS. Beyond its analgesic effects, WBC appears to enhance broader domains of patient well-being, including perceived health status, psychological resilience, quality of life, and ability to perform activities of daily living, all without the adverse effects commonly associated with pharmacological treatments or the burden of invasive procedures. This favorable safety and tolerability profile positions WBC as a promising adjunct or alternative to conventional pain therapies, especially in patients who are refractory to standard approaches or wish to avoid long-term medication use. These preliminary findings strongly support the need for further investigation through larger, controlled clinical trials to rigorously evaluate the efficacy, optimal protocols, and mechanisms of action of WBC in CRPS. Future studies should involve larger sample sizes, include control groups, and implement long-term follow-up to determine the sustained effects of WBC and its specific contribution to rehabilitation outcomes. This present case study is innovative because it demonstrates the potential benefits of WBC, thus widening the rehabilitative options for CRPS patients.

## 5. Conclusions

In conclusion, the findings from this case study suggest that WBC is a safe, well-tolerated, non-invasive, intervention for modulating pain and improving overall functional outcomes and psychosocial well-being in patients affected by CRPS. The marked improvements observed across multiple domains, including pain intensity, disability, mood, sleep quality, and functional mobility, highlight the potential of WBC not only as a symptomatic treatment but as a restorative approach capable of enhancing quality of life and daily functioning without the side effects commonly associated with pharmacological therapies. Its favorable safety profile, ease of application, and systemic effects make WBC particularly attractive in the context of chronic pain conditions where conventional therapies often fall short or are poorly tolerated.

Further research with larger sample sizes and longer follow-up periods is warranted to confirm these findings and explore the long-term benefits of WBC in the management of CRPS and other complex pain syndromes characterized by inflammation, central sensitization, and psychological burden.

## Figures and Tables

**Table 1:** Summary of psychosocial outcomes before and after the WBC cycle.

Outcome and Range	Pre	Post	Δ%
SF-36			
*Physical functioning*	30%	35.7%	+5.7%
*Role limitations due to physical health*	0%	25%	+25%
*Role limitations due to emotional problems*	67%	100%	+33%
*Energy/fatigue*	44%	70%	+26%
*Emotional well-being*	44%	72%	+28%
*Social functioning*	75%	75%	0
*Pain*	50%	60%	+10%
*General health*	25%	55%	+30%
WHO-5 (0–100)	36	68	+88.9%
PSQI (0–21)	18	10	−44.4%
FSS (9–63)	50	27	−46%
BDI (0–63)	12	10	−16.7%
STAI 1 (20–80)	47	34	−27.7%
STAI 2 (20–80)	51	43	−15.7%

Table 1 abbreviation list: BDI: Beck Depression Inventory; FSS: Fatigue Severity Scale; PSQI: Pittsburgh Sleep Quality Index; SF-36: 36-Item Short-Form Health Survey; STAI: State–Trait Anxiety Inventory; WHO-5: World Health Organization–Five Well-Being Index.

**Table 2:** Changes in pain intensity, pain-related disability, and pain-related cognition before and after the WBC intervention.

Outcome and Range	Pre	Post	Δ%
PRI SF-MPQ (1–78)	58	28	−51.7%
VAS (0–10)	9	3	−66.7%
PNRS (0–10)	10	3	−70%
PDI (0–70)	45	38	−15.6%
PCS (0–52)	34	9	−73.5%

Table 2 abbreviation list: PCS: Pain Catastrophizing Scale; PDI: Pain Disability Index; PNRS: Pain Numerical Rating Scale; PRI SF-MPQ: Pain Rating Index of the Short-Form McGill Pain Questionnaire; VAS: Visual Analogue Scale.

**Table 3:** Changes in functional mobility and lower-limb motor performance before and after the WBC intervention.

Outcome and Range	Pre	Post	Δ%
TUG (seconds)	11.55	7.88	−31.8%
Chair Stand Test (reps)	8	10	+25.0%

**Table 4:** Changes in joint range of motion and muscle strength before and after the WBC intervention.

Measure	Side	Pre	Post	Δ%
Ankle Dorsiflexion AROM (°)	R	15	25	+66.7%
	L	15	25	+66.7%
Ankle Plantarflexion AROM (°)	R	25	40	+60%
	L	35	40	+14.3%
Knee MRC Flexion	R	3	4	+33.3%
	L	3	4	+33.3%

Table 4 abbreviation list: AROM: Active Range of Motion; L: Left; MRC: Medical Research Council scale; R: Right.

**Table 5:** Skin temperature changes at the ankle level across the 15-session WBC protocol.

Session	Right Ankle Pre (°C)	Right Ankle Post (°C)	Δ Right (°C)	Left Ankle Pre (°C)	Left Ankle Post (°C)	Δ Left (°C)
1	35.2	26.9	−8.3	32.0	22.3	−9.7
2	34.6	24.3	−10.3	33.0	24.0	−9.0
3	32.1	24.1	−8.0	30.3	24.9	−5.4
4	34.5	26.9	−7.6	32.3	26.3	−6.0
5	35.2	26.7	−8.5	35.2	25.0	−10.2
6	34.3	23.7	−10.6	34.2	23.9	−10.3
7	33.4	23.8	−9.6	33.0	23.5	−9.5
8	34.8	24.4	−10.4	34.0	24.1	−9.9
9	34.1	23.7	−10.4	32.4	23.4	−9.0
10	34.2	25.2	−9.0	34.0	24.4	−9.6
11	34.6	23.8	−10.8	34.3	23.9	−10.4
12	34.8	25.5	−9.3	34.5	24.7	−9.8
13	33.9	26.4	−7.5	33.5	23.5	−10.0
14	33.6	23.7	−9.9	33.4	24.0	−9.4
15	33.4	25.7	−7.7	33.0	23.2	−9.8

**Table 6:** Magnetic resonance imaging findings of the knee and ankle across pre- and post–WBC timepoints.

Site	MRI Date	Key Findings
Right Knee	August 2024	Meniscal extrusion, cartilage thinning, mild edema, early degenerative changes.
	7 July 2025 (Pre-WBC)	Meniscal radial tear (medial), early tibiofemoral chondropathy (Grade II), mild synovial reaction, no significant effusion.
	19 August 2025 (Post-WBC)	Stable meniscal and ligamentous findings. Notably reduced synovial reaction, absence of joint effusion, and no new degenerative signs.
Right Ankle	August 2024	Subchondral bone edema (cystic appearance), dorsal soft tissue edema, signs of algodystrophy.
	7 July 2025 (Pre-WBC)	Edema in periastragalic region and sinus tarsi, signs of chondropathy and joint space narrowing.
	19 August 2025 (Post-WBC)	Reduction in soft tissue edema, improved subchondral bone appearance, no progression of arthrosis, stable cartilage.

## Data Availability

The data presented in this study are available within the article.
